# The Development of Cardiac Rehabilitation in China: Current Status and Future Perspectives

**DOI:** 10.31083/j.rcm2507233

**Published:** 2024-06-27

**Authors:** Siwei Li, Hao Xu

**Affiliations:** ^1^Rehabilitation Medical Center, Beijing Jingmei Group General Hospital, 102300 Beijing, China; ^2^National Clinical Research Center for Chinese Medicine Cardiology, Xiyuan Hospital, China Academy of Chinese Medical Sciences, 100091 Beijing, China

**Keywords:** China, cardiac rehabilitation, historical development, clinical mode

## Abstract

In recent years, significant progress has been made in China in the field of cardiac rehabilitation by raising awareness among the Chinese public, scholars, hospitals, and government regarding the substantial benefits of cardiac rehabilitation/secondary prevention of cardiovascular diseases. Cardiac rehabilitation encompasses a comprehensive intervention strategy for cardiovascular diseases, integrating multiple disciplines, such as cardiology, sports medicine, rehabilitation medicine, nutriology, psychology, behavioral medicine, and preventive medicine. Standardized and systematic cardiac rehabilitation therapy can help patients with cardiovascular diseases restore their physical and mental health and reduce the risk of recurrences and deaths from cardiovascular diseases. This article provides an overview of the historical development, existing clinical practice modes, and the latest clinical research findings on cardiac rehabilitation, focusing on the current clinical modes and clinical research results of cardiac rehabilitation in China. It aims to offer a systematic perspective for international peers to know and understand cardiac rehabilitation in China, along with an objective analysis and future prospects for advancing this field.

In the 1980s, China, which had just embarked on its reform and opening-up policy, witnessed a new wave of global engagement. Chinese doctors, who had also been liberated in their thinking, shifted their focus to the international stage. One area that caught their attention was cardiac rehabilitation, a field that had only emerged 30 years prior and entered the realm of Chinese medicine during this period. Following in the footsteps of their international counterparts, the pioneers of cardiac rehabilitation in China embarked on a journey of exploration, overcoming denial, skepticism, and trial and error. Gradually, they established, improved, and enriched the theoretical and practical aspects of cardiac rehabilitation in China, formulating a wealth of valuable experience and viable models. This article provides an overview of the development and current status of cardiac rehabilitation in China and discusses its future trends.

## 1. Overview of the History of Cardiac Rehabilitation in China

### 1.1 Enlightenment Stage

As early as the 1970s, some visionary Chinese medical experts recognized the importance of cardiac rehabilitation. However, it was not until 1981, when a delegation from the World Health Organization (WHO) visited China to discuss cardiac rehabilitation, that the concept began to be truly understood by the Chinese medical community. In the same year, Dr. Wu Yingkai, a renowned Chinese medical scientist and pioneer of thoracic and cardiovascular surgery, published a review article entitled “Research work on the importance of rehabilitation treatment for cardiovascular diseases” in the influential academic journal “*Chinese Journal of Cardiology*” [1]. He was the first to publicly propose the concept of cardiac rehabilitation for treating cardiovascular diseases in China. Subsequently, medical teams from renowned hospitals, such as Guangdong Provincial People’s Hospital, Xiangya Hospital, Hebei Provincial People’s Hospital, Zhejiang Hospital, and Peking University Third Hospital embarked on clinical practice and research exploration in cardiac rehabilitation. During this period, cardiac rehabilitation was mainly led by the Department of Rehabilitation Medicine, with participation from the cardiovascular discipline.

In 1990, the Chinese Association of Rehabilitation Medicine Cardiovascular Disease Professional Committee was officially established, making it the first academic organization dedicated to cardiac rehabilitation in China. The committee has subsequently formulated and promulgated various standards and reference programs, such as the “Interpretive Criteria of Grated Exercise Test Results (Draft)” [2], the “Reference scheme of Chinese program for AMI cardiac rehabilitation” [3], and the “Chinese rehabilitation program after percutaneous coronary intervention (Trial version)” [4]. These criteria and guidelines have standardized and guided clinical practices in cardiac rehabilitation, promoting the development of cardiac rehabilitation in China. However, at that time, most of the experts involved in cardiac rehabilitation were from the field of rehabilitation medicine, and these standards and guidelines did not significantly impact cardiovascular doctors.

It is worth mentioning that interventional diagnostic and treatment techniques for cardiovascular diseases were also introduced in China during this period. Thus, these techniques developed rapidly with the support of experts, capital investment, and national recognition. However, concerns about cardiovascular events induced by exercise still led some experts to question the safety of cardiac rehabilitation. Hence, cardiac rehabilitation did not receive much attention from the academic community and government agencies in the field of cardiovascular medicine for over a decade. Consequently, although cardiac rehabilitation in China started around the same time as cardiac interventional techniques, its development significantly lagged behind the latter. During this period, the actual clinical practice of cardiac rehabilitation gradually disappeared within two decades, with only a few hospitals retaining limited technical expertise in cardiac rehabilitation to maintain disciplinary integrity and diversity. Moreover, these efforts mostly remained at the research level [5].

### 1.2 Exploratory Stage

As the new century approached, thanks to the relentless efforts of pioneering experts in the field of cardiac rehabilitation in China, cardiac rehabilitation began to receive attention and recognition from cardiovascular experts, scholars, and government agencies. Some research projects even received funding support at the national level. Subsequently, Chinese cardiac rehabilitation entered a new stage. During this period, several hospitals gradually adopted a cardiac rehabilitation model led by the Department of Cardiovascular Medicine, with cardiovascular physicians leading in clinical practice.

Over the following decade, China experienced a notable rise in the number of experts and scholars specializing in cardiac rehabilitation. Their proactive efforts played a crucial role in advancing the development of cardiac rehabilitation within the country. They diligently organized many academic forums and technical training courses to promote and popularize the fundamental principles and concepts of cardiac rehabilitation.

### 1.3 Rapid Expansion Stage

In 2012, renowned cardiovascular expert Dr. Hu Dayi introduced cardiac rehabilitation into the field of cardiovascular medicine and disease treatment systems, proposing the concept of the “Five Prescriptions for Cardiac Rehabilitation”. In 2013, the Chinese Society of Cardiology, in collaboration with the Chinese Association of Rehabilitation Medicine Cardiovascular Disease Professional Committee and other societies, jointly released the “Expert consensus on coronary heart disease rehabilitation and secondary prevention in China” [6]. It divides the standard mode of cardiac rehabilitation into three phases: The in-hospital rehabilitation phase (Phase I), the early outpatient or clinic rehabilitation phase (Phase II), and the community/home long-term rehabilitation phase (Phase III). At the same time, this consensus, for the first time, lists the five major prescriptions for cardiac rehabilitation as the recommended program in China. These prescriptions encompass exercise, nutrition, psychology, smoking cessation, and medication. This comprehensive model, which combines international prevention and rehabilitation concepts with Chinese characteristics, greatly propelled the development of cardiac rehabilitation in China. Subsequently, multiple expert consensus statements related to cardiac rehabilitation were published [7, 8, 9, 10, 11]. In 2015, the Chinese Association of Rehabilitation Medicine Cardiovascular Disease Professional Committee released the first edition of the “Guidelines for Cardiac Rehabilitation and Secondary Prevention in China” [12, 13] (last updated in 2018), which recommended the Five Prescriptions for Cardiac Rehabilitation. In 2016, the National Center for Cardiovascular Diseases in China published the “Expert Consensus on Integrated Chinese and Western Medicine Phase I Cardiac Rehabilitation” [14]. Building upon the Five Prescriptions, this consensus introduced Chinese traditional medicine diagnosis and treatment methods and innovative exercise rehabilitation approaches, proposing the “Jiu-jiu (Nine Long-Term in Chinese) Rehabilitation” model for cardiac rehabilitation. The model expanded the Five Prescriptions to include respiratory exercise, nutrition, exercise, sleep, pain management, psychology, smoking cessation, secondary prevention medication, and traditional Chinese medicine. These nine domains require long-term intervention and management, laying an important foundation for the localization of cardiac rehabilitation in China. In 2017, the National Center for Cardiovascular Diseases in China again published the “Clinical Pathways for Cardiovascular Disease Prevention and Rehabilitation” [15]. The “Jiu-jiu Rehabilitation” model was officially incorporated into the clinical pathway for cardiac rehabilitation, marking the beginning of the localization process for cardiac rehabilitation in China.

**Fig. 1.**
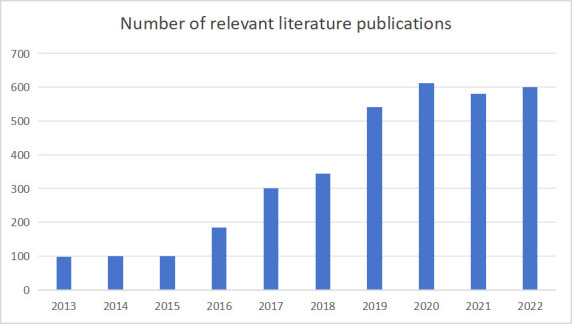
**Number of relevant literature publications on cardiac rehabilitation in China from 2013 to 2022.** Nevertheless, considering China’s large population, the density of hospitals offering cardiac rehabilitation is relatively low at 13.2 per 100 million people, which is much lower than in developed countries. Additionally, due to the vast territorial expanse and complex geographic and economic conditions of China, most cardiac rehabilitation centers are concentrated in the economically developed southeastern coastal regions, while the northwest and southwest regions are relatively underserved [18]. Hence, there is still ample room for the development of cardiac rehabilitation in China.

Since then, cardiac rehabilitation centers have sprung up across China. As of 2017, over 500 cardiac rehabilitation centers have been established nationwide. In 2018, the Chinese Association of Rehabilitation Medicine Cardiovascular Disease Professional Committee presented the findings of a survey on the current status of cardiac rehabilitation in China at the American College of Cardiology 67th Annual Scientific Session and Expo (ACC2018). The data revealed that 22% of hospitals nationwide implemented cardiac rehabilitation programs, with 13% offering Phase I cardiac rehabilitation, 17% offering Phase II cardiac rehabilitation, and 8% offering both Phase I and Phase II cardiac rehabilitation [16]. Furthermore, a survey conducted in 2021 indicated that among the 76 tertiary hospitals in 10 Chinese provinces, over 50% of the hospitals were providing Phase I cardiac rehabilitation. The implementation methods varied, with over 97% of the hospitals offering cardiopulmonary exercise testing among those providing Phase I cardiac rehabilitation [17]. These data suggest that healthcare professionals and hospital administrators in China have recognized the value of cardiac rehabilitation for patients. During this period, there has been an increasing number of clinical research studies on cardiac rehabilitation. A search using “cardiac rehabilitation” as a keyword in the largest academic paper database in China, the China National Knowledge Infrastructure (CNKI), showed that the number of related publications was less than 100 per year between 2013 and 2015. However, since 2016, the number of publications has increased exponentially, stabilizing at around 600 per year from 2020 onwards (Fig. 1, Ref. [18]).

## 2. Current Status of Cardiac Rehabilitation in China

Despite the strict control measures to prevent the spread of the novel coronavirus in the past three years, which have had some adverse effects on the development of cardiac rehabilitation in China, overall, the progress of cardiac rehabilitation in China has been extremely rapid over the past decade. On the one hand, this is manifested by a significant increase in cardiac rehabilitation centers and the publication of related research papers, as mentioned earlier. On the other hand, it is also reflected in the deepening awareness of cardiac rehabilitation among society, patients, the medical community, and government agencies.

After more than 40 years of development, China has explored specific patterns in the clinical practice of cardiac rehabilitation and has achieved certain accomplishments in clinical research. This section will elaborate on these two aspects.

### 2.1 Clinical Practice Patterns of Cardiac Rehabilitation in China

A survey conducted among healthcare professionals in Chinese hospitals indicates that promoting the development of cardiac rehabilitation in China relies on training specialized cardiac rehabilitation personnel, obtaining support from the government and hospitals, establishing multidisciplinary collaboration teams, and establishing a referral system for cardiac rehabilitation [19]. This highlights the crucial role of localized cardiac rehabilitation models in developing cardiac rehabilitation in China. Currently, there are primarily five main clinical practice patterns for cardiac rehabilitation in China.

#### 2.1.1 Integrated Hospital Rehabilitation Department or Subspecialty Model

Under the rehabilitation department (hospital), there is a subspecialty of cardiac rehabilitation that is linked to various clinical departments. Cardiac rehabilitation parallels other subspecialties such as stroke rehabilitation, orthopedic surgery rehabilitation, and oncology rehabilitation, forming a comprehensive rehabilitation clinical department led by rehabilitation medicine experts. Its main role is to assist patients in implementing Phase II cardiac rehabilitation programs.

#### 2.1.2 Cardiac Rehabilitation Center Model

This model involves the establishment of an independent cardiac rehabilitation center, cardiac rehabilitation department, or even an affiliated cardiac rehabilitation hospital in addition to the integration of traditional cardiology and cardiovascular surgery. Such models are usually found in cardiovascular specialty hospitals or comprehensive hospitals with a cardiovascular advantage. Cardiovascular specialists primarily lead them in conducting clinical practices for cardiac rehabilitation. Their teams are based on the cardiovascular specialty, allowing them to assist patients in implementing Phase I cardiac rehabilitation plans within the hospital and Phase II plans in outpatient settings.

#### 2.1.3 Integration of Cardiovascular Medicine and Cardiac Rehabilitation Model

This model is more common in comprehensive hospitals where cardiovascular specialties are not prominent. The cardiac rehabilitation team is affiliated with the traditional cardiology and cardiovascular surgery departments and is also led by cardiovascular specialists who conduct cardiac rehabilitation clinical practices. Similarly, due to the team’s affiliation with the cardiovascular specialty, they have a significant advantage in assisting patients in implementing Phase I cardiac rehabilitation plans within the hospital. However, compared to the independent cardiac rehabilitation center model, this model generally faces difficulties establishing separate cardiac rehabilitation outpatient services, resulting in some disadvantages in helping patients with Phase II outpatient cardiac rehabilitation plans. They often rely on remote home-based Phase II cardiac rehabilitation guidance to help patients complete their rehabilitation plans.

#### 2.1.4 Hospital-Led Home Cardiac Rehabilitation Model

Generally, partial Phase II and all Phase III cardiac rehabilitation programs should be conducted at home. Therefore, home cardiac rehabilitation is crucial for patients’ comprehensive recovery. However, due to factors such as limited cognition, skills, and medical resources, most patients have weaker adherence to cardiac rehabilitation plans after leaving the hospital compared to when they were in the hospital. In response, some hospitals develop home cardiac rehabilitation plans for patients and continue to provide care outside the hospital setting. This may include timely phone reminders, home visits, remote cardiac rehabilitation guidance, and other methods to monitor the implementation of home cardiac rehabilitation and guide patients on executing their cardiac rehabilitation and addressing related questions. These models are often an extension of the first three models but are still less developed in China than various in-hospital cardiac rehabilitation models.

#### 2.1.5 The Expansion of Functions in Comprehensive Hospitals or Cardiovascular Disease Specialized Hospitals’ Health Management Centers

Comprehensive management and services are provided to high-risk populations and patients with subclinical conditions. This model is relatively niche in China.

However, regardless of the model, in the process of actual clinical practice, most hospitals implementing cardiac rehabilitation still face common issues, such as a lack of specialized talent, confusion in the roles of medical staff, doctors performing the work of exercise therapists, nurses assuming multiple roles, and exercise therapists not being involved in cardiac rehabilitation. Additionally, comprehensive assessment in cardiac rehabilitation clinical operations is often neglected alongside a lack of comprehensive individualized cardiac rehabilitation prescriptions; however, even if assessments are conducted, the prescriptions remain generic without addressing specific patient issues or treatment goals, while also lacking supervision of prescription implementation. There is also a lack of evaluation of quality control data in the cardiac rehabilitation process. In fact, cardiac rehabilitation has not yet formed a true professional discipline in China [20]. Therefore, some scholars believe that [21] the current focus of cardiac rehabilitation in China should be establishing cardiac rehabilitation centers and popularizing the significance of cardiac rehabilitation. Compared to developed countries, China has not fully implemented standardized clinical practices in cardiac rehabilitation, and many rehabilitation centers lack facilities, equipment, staffing, and appropriate standards for cardiac rehabilitation assessment and treatment fees. These factors significantly hinder the positive development of cardiac rehabilitation and make it difficult to demonstrate its medical and social values.

### 2.2 Current Status of Clinical Research in Cardiac Rehabilitation in China

Expanding to the theoretical and clinical research levels, cardiac rehabilitation in China has become more standardized and advanced, aligning with international peers in the field. Chinese experts and professional academic organizations in cardiac rehabilitation have researched and summarized findings and developed numerous clinical practice consensus statements, guidelines, clinical pathways, and other normative guidance documents. These efforts have greatly promoted the implementation of cardiac rehabilitation in clinical practice.

Clinical research in cardiac rehabilitation in China has consistently focused on cardiovascular disease patients, such as those with chronic heart failure, coronary heart disease, acute myocardial infarction, percutaneous coronary intervention (PCI), and coronary artery bypass graft surgery. The cardiac rehabilitation programs primarily revolve around exercise therapy. Moreover, the study of exercise training methods in cardiac rehabilitation is a research focus for Chinese scholars. Among them, the research on traditional Chinese medicine diagnostics and treatment and exercise rehabilitation methods showcases the localization characteristics of Chinese researchers. At the same time, research on remote home-based rehabilitation methods has been increasingly valued, and a batch of related studies on auxiliary cardiac rehabilitation technologies has emerged. In addition, Chinese cardiac rehabilitation nursing experts have translated and validated influential international cardiac rehabilitation assessment tools, such as the Cardiac Rehabilitation Barriers Scale (CRBS) and the Information Needs in Cardiac Rehabilitation (INCR), effectively providing more options for personalized assessment and care for Chinese patients undergoing cardiac rehabilitation.

In recent years, the following sections have formulated the main research hotspots.

#### 2.2.1 Physical Ischemic Training (PIT)

Ischemic preconditioning training is a recently proposed exercise training method in cardiac rehabilitation, primarily aimed at improving blood supply to the ischemic area of the myocardium by utilizing the self-protective mechanism of myocardial ischemia. PIT can increase the expression of endothelial nitric oxide synthase mRNA and protein, promote the elevation of vascular endothelial growth factor, endothelial progenitor cells, and nitric oxide (NO) levels, and increase NO-mediated mobilization of bone marrow endothelial progenitor cells, thereby improving local ischemic myocardial capillary density and coronary collateral circulation flow [22]. Animal experiments have shown that PIT can protect rabbit endothelial function and slow the formation of atherosclerotic plaques [23]. It can also increase skeletal muscle contractile endurance in rabbits by altering skeletal muscle cell oxidative potential and fiber distribution, suggesting that PIT benefits the exercise capacity of patients with coronary heart disease [24]. It should be noted that PIT may induce recurrent ischemic myocardial injury, meaning its mechanisms of action and safety need further research for confirmation.

#### 2.2.2 High-Intensity Interval Training (HIIT)

To reduce exercise-related risks, previous guidelines have mostly recommended moderate-intensity continuous training (MCT) as aerobic exercise for coronary heart disease patients in cardiac rehabilitation programs. However, with advancements in rehabilitation techniques, recent research has indicated that HIIT has superior cardiac rehabilitation effects compared to MCT in patients with coronary heart disease [25, 26]. HIIT is a form of cardiac rehabilitation exercise training that combines short-duration, high-intensity exercise training with low-intensity exercise training or rest intervals, with two high-intensity training periods followed by one low-intensity training or rest period. Studies have shown that healthy adults undergoing HIIT have lower cardiovascular reactivity and myocardial oxygen consumption [27]. Animal experiments have demonstrated that HIIT significantly increases the expression of mechanical growth factor (MGF)-mitogen-activated protein kinase kinase (MEK)1/2-extracellular signal-regulated kinase (ERK)1/2 in infarcted myocardium and skeletal muscle, reduces infarct size, improves cardiac function, and alleviates post-infarction skeletal muscle loss [28]. Both HIIT and MCT can alleviate myocardial mitochondrial dysfunction after myocardial infarction in rats, with HIIT being more advantageous than MCT [29].

#### 2.2.3 Traditional Chinese Medicine Diagnosis and Treatment in Exercise Rehabilitation

Traditional Chinese medicine (TCM) has been implemented for thousands of years in China, and the general public has a high acceptance of TCM therapies. Therefore, TCM also plays a unique role in cardiac rehabilitation in China. Traditional therapies such as acupuncture and herbal patches have been utilized in cardiac rehabilitation diagnosis and treatment. Research has shown that a specific acupuncture technique can significantly reduce the B-type natriuretic peptide (BNP) levels and improve heart function in heart failure patients assessed by New York Heart Association (NYHA) classification after 14 days of treatment [30]. Another acupoint patching method can reduce the frequency and symptoms of stable angina attacks in patients [31]. In addition, TCM includes various traditional exercise rehabilitation methods known as “Dao Yin”, including Six Healing Sounds, Eight Section Brocade, Tai Chi, Five Animal Frolics, and Yi Jin Jing. In recent years, numerous studies in China have demonstrated the positive effects of these traditional exercise rehabilitation methods on improving exercise endurance, enhancing cardiopulmonary function, and other aspects [32, 33, 34, 35, 36, 37, 38, 39] (Table 1).

**Table 1.** **List of research literature on cardiac rehabilitation using TCM exercise rehabilitation methods**.

TCM exercise rehabilitation methods	Study group	Sample size	Conclusion
Liuzijue	CHF	90	Improved exercise endurance and cardiopulmonary function
Sitting Baduanjin	CABG	245	Improved cardiopulmonary function and raised detection rate of anaerobic threshold
	PCI after AMI	150	Improved heart function and quality of life
TaiChi(Ji)Quan	HF after MI	200	Improved heart function, exercise endurance, and quality of life
	CAHD	24	Good for heart function
Wuqinxi	CHF	60	Improved clinical symptoms and cardiac function
	CR patients	80	Improved the heart function and the quality of daily life
Yijinjing	HFrEF and HFmrEF	70	Safe and effective; improved exercise tolerance, cardiopulmonary reserve function, and the quality of life

Note: CHF, congestive heart failure; HF, heart failure; CABG, coronary angioplasty bypass grafting; PCI, percutaneous coronary intervention; AMI, acute myocardial infarction; MI, myocardial infarction; CAHD, coronary atherosclerotic heart disease; CR, cardiovascular rehabilitation; HFrEF, heart failure with reduced ejection fraction; HFmrEF, heart failure with mildly reduced ejection fraction; TCM, Traditional Chinese medicine.

#### 2.2.4 Remote Home Cardiac Rehabilitation Devices

China is also embarking on the exploration of remote home cardiac rehabilitation on a domestic level. Due to its implementation not being restricted to hospitals, remote home rehabilitation offers personalized, convenient, and cost-effective features, providing an alternative for patients unable to participate in in-hospital cardiac rehabilitation. In the past three years, the impact of epidemic control measures has led to difficulties in transportation and increased challenges in accessing medical services, thereby affecting the effective implementation of traditional in-hospital cardiac rehabilitation. On the other hand, it has significantly facilitated the development of remote home-based cardiac rehabilitation and raised higher demands for its implementation.

Remote home cardiac rehabilitation often relies on portable devices, such as fitness trackers, which can be controlled and utilized through apps installed on mobile phones, tablets, and computers. In an ideal scenario, remote home-based cardiac rehabilitation should naturally follow and serve as the main component of Phase I and II cardiac rehabilitation conducted in hospitals and outpatient settings, leading into Phase III cardiac rehabilitation. However, the practical application of technology-assisted cardiac rehabilitation in China is still in its early stages. In 2019, a study on technology-assisted cardiac rehabilitation in Wuhan [40] randomly selected 78 coronary heart disease patients with concomitant abdominal obesity and divided them into two groups: Conventional cardiac rehabilitation and technology-assisted cardiac rehabilitation. The technology-assisted cardiac rehabilitation program assisted patients in completing an entire 12-week cardiac rehabilitation program, transitioning from in-hospital to outpatient care. The results showed that technology-assisted cardiac rehabilitation had significant effects in improving physical activity, promoting a healthy lifestyle, smoking cessation, and reducing body mass index and waist circumference. However, in most cases, such research in China is limited to Phase III home-based cardiac rehabilitation. In a previous study [41], 200 cardiac patients were randomly divided into a home cardiac rehabilitation intervention group supervised by Xiaomi fitness trackers and a control group for self-management. The results showed that the peak ${\mathrm{VO}}_{2}$ and peak oxygen pulse in the group using fitness trackers were higher than those in the control group. Another study [42] randomly assigned 124 myocardial infarction patients to an observation group monitored by fitness trackers and a control group without monitoring. The results showed that at the 6-month follow-up, the adherence in the observation group improved, and the rehospitalization rate decreased. Research has proven that portable remote home cardiac rehabilitation devices such as fitness trackers indeed help motivate patients to exercise, monitor and record their physiological and training data, promote the development of exercise habits, and, ultimately, improve cardiopulmonary function. However, overall, existing devices have issues with data accuracy, significant interference during usage, and the inability to monitor certain physiological data in real-time, which still do not fully meet the needs of remote home cardiac rehabilitation. Therefore, domestic and foreign manufacturers are continuously improving technology and optimizing algorithms to better adapt to the training needs of remote cardiac rehabilitation in the future. At the same time, technology-assisted cardiac rehabilitation outside the hospital is also continuously extending to in-hospital cardiac rehabilitation, aiming to achieve an ideal integration of in-hospital and out-of-hospital cardiac rehabilitation, enhancing the compliance, continuity, and effectiveness of cardiac rehabilitation both inside and outside the hospital.

#### 2.2.5 Auxiliary Cardiac Rehabilitation Technologies

Enhanced External Counter Pulsation (EECP) and Extracorporeal Cardiac Shock Wave Therapy (CSWT) have been among the recent research hotspots in cardiac rehabilitation in China.

EECP is an auxiliary cardiac rehabilitation technology that improves blood circulation through external forces and has been used to treat ischemic heart disease since the 1970s [43]. In 2013, the European Society of Cardiology guidelines recommended EECP as an adjunctive therapy for cardiac rehabilitation [44]. In 2014, the “Cardiovascular Disease Rehabilitation Prescription—Enhanced External Counter Pulsation Application International Expert Consensus” in China recommended EECP as an adjunctive therapy for cardiac rehabilitation [45]. A clinical trial study [46] confirmed that EECP effectively reduces the frequency of angina attacks in patients with coronary heart disease, decreases ST-segment depression, and improves left ventricular ejection fraction. Another study [47] showed that EECP effectively improves short-term hemodynamic conditions in patients with unstable angina after PCI and has a high level of safety, suggesting that EECP is safe and effective for coronary heart disease patients after PCI.

CSWT is a relatively new and emerging approach in the field of cardiac rehabilitation. It is a non-invasive method that utilizes the effects of ultrasound on the myocardium to generate various directional forces and vibration effects for vascular reconstruction, offering certain advantages in cardiac rehabilitation. Studies have indicated that integrin-linked kinase (ILK) expression may be one of the key regulatory factors in promoting collateral formation by CSWT and is also a critical factor in a series of biomechanical effects, potentially becoming a breakthrough in the study of the biomechanical mechanisms involved in CSWT [48]. Furthermore, CSWT has a protective effect against ischemia/hypoxia-induced cell apoptosis, and its mechanism of action may be related to the activation of the protein kinase B pathway, upregulation of Bcl-2 expression, and downregulation of pro-apoptotic molecules Bax and caspase-3 [49]. However, a study [50] found that CSWT had no significant effect on hemodynamic parameters, serum cardiac troponin I, or left ventricular function in rats, and it did not induce myocardial inflammatory response or fibrotic changes. Electron microscopy observation also showed no significant additional damage to the myocardial ultrastructure. Therefore, despite the relatively short application and research time of CSWT in cardiac rehabilitation for coronary heart disease patients, it has high safety and certain value for further promotion and application [51].

Although cardiac rehabilitation in China started relatively late, clinical researchers have always kept up with international trends and demonstrated certain characteristics in localizing traditional Chinese medicine. Overall, China has had limited collaborative research with other countries in the field of cardiac rehabilitation. The network map of international collaborative research in cardiac rehabilitation [52] shows that China has fewer collaborative relationships with other countries in cardiac rehabilitation research than developed countries. Additionally, the number of published articles, citation frequency, and quality of literature in Chinese cardiac rehabilitation research lag behind international peers. There is still a need for in-depth development and exploration of clinical research on cardiac rehabilitation in China. Indeed, Chinese cardiac rehabilitation research should strengthen its efforts in promoting local research and utilize research data to advocate for support from the government, insurance providers, and patients.

#### 2.2.6 Translation and Validity Testing of Chinese Versions of Cardiac Rehabilitation Nursing Assessment Tools

Cardiac rehabilitation is a comprehensive system involving multiple disciplines, such as medicine, rehabilitation, nutrition, psychology, and nursing. As important facilitators in the cardiac rehabilitation process, nursing professionals are involved in developing medical rehabilitation plans, directly guiding and supervising the implementation of patient rehabilitation, and continuously assessing, formulating, implementing, providing feedback, revising, and re-implementing in a dynamic cycle throughout the rehabilitation process. Indeed, using tools such as scales in nursing assessments can standardize the assessment process and content, ensuring the quality of nursing assessments.

Both domestic and international scholars have developed a series of assessment tools from perspectives such as the patients’ objective impairments and subjective psychological barriers. Chinese cardiac rehabilitation nursing experts, in collaboration with international colleagues, have conducted translation, validation, and reliability testing studies on influential cardiac rehabilitation nursing assessment tools worldwide. The scales primarily include the CRBS [53, 54] and the INCR scale [55, 56] for assessing objective impairment factors in patients. Additionally, to assess the subjective perception barriers in patients, the scales mainly include the Cardiac Rehabilitation Inventory (CRI) [57], the Cardiac Exercise Self-Efficacy Instrument (CESEI), and the Cardiac Diet Self-Efficacy Instrument (CDSEI) [58, 59, 60]. The translation and related research of these cardiac rehabilitation nursing assessment scales provide more options for personalized assessment and nursing care for Chinese patients undergoing cardiac rehabilitation. Researchers can choose suitable tools for assessing appropriate populations based on different purposes or develop cardiac rehabilitation nursing assessment tools suitable for China based on the national conditions and sociocultural background.

## 3. Future Trends in Cardiac Rehabilitation in China

China’s enlightenment in cardiac rehabilitation lagged behind for more than 30 years. However, over 40 years of arduous struggle and exploration, with several generations of Chinese scholars engaged in cardiac rehabilitation and secondary prevention, have continuously narrowed and bridged the gap.

An ideal cardiac rehabilitation center in China should have a cardiac-cultured cardiovascular physician at the core, with exercise therapists and rehabilitation nurses as key members of the cardiac rehabilitation team. It should also be equipped with nutritionists, psychologists, pharmacists, and traditional Chinese medicine practitioners for collaboration. This center should be able to personalize cardiac rehabilitation assessments, develop individualized cardiac rehabilitation prescriptions, implement comprehensive cardiac rehabilitation plans, and evaluate and perform reasonable adjustments and improvements based on the clinical effects, behavioral outcomes, health effects, and service outcomes of patients’ cardiac rehabilitation.

Based on this foundation, a complete cardiac rehabilitation model should gradually form, relying on technologies such as telemedicine and wearable artificial intelligence devices. It should involve comprehensive collaboration among hospitals, communities, and families, covering the entire cardiac rehabilitation process from Phase I to Phase III. Concurrently with standardized cardiac rehabilitation, high-quality clinical research should be conducted, organizing multicenter, large-sample, randomized controlled clinical trials and reporting clinical research results according to international standards.

China will have an increasing number of hospitals incorporating cardiac rehabilitation into their daily treatment, and there will be a growing emergence of cardiac rehabilitation centers. With the continuous advancement of clinical practice, cardiac rehabilitation in certain developed regions of China has already taken on the rudimentary form of this ideal. Based on this foundation, cardiac rehabilitation in China continuously makes efforts towards localization. Traditional Chinese medicine techniques such as herbal medicine, poultices, acupuncture, guidance, and qigong are being incorporated into cardiac rehabilitation programs. Additionally, there is a positive and open attitude towards developing, introducing, and utilizing various suitable cardiac rehabilitation technologies from other sources.

Currently, scholars have called [61] for standardized research on traditional exercise prescriptions in China, including data collection on action standards and quantification of exercise intensity. Particularly, data on exercise intensity levels in cardiovascular disease populations are needed to make traditional exercise forms more targeted, safe, and widely applicable. Active research is being conducted on the physiological mechanisms of traditional exercise forms in China. Therefore, it is necessary to understand the impact mechanisms of traditional exercise on organ function levels and explore the impact mechanisms of traditional exercise at the molecular biology level. Specifically, it is important to elucidate the basis of exercise physiology and mechanisms of “mind–body exercise” in traditional Chinese exercise, providing strong scientific evidence for the role and advantages of traditional exercise in the cardiovascular system. In addition, the development of new technologies, such as artificial intelligence (AI)-assisted cardiac rehabilitation, will positively impact cardiac rehabilitation in China. This includes prediction and assessment, program development, rehabilitation training execution and feedback, supervision and management, research, and development. The future of cardiac rehabilitation in China will undoubtedly be a smart and innovative model that integrates localized rehabilitation intervention methods based on advanced international prevention and rehabilitation concepts.

## References

[1] Wu YK (1981). Research work on the importance of rehabilitation treatment for cardiovascular diseases. *Chinese Journal of Cardiology*.

[2] Liu JS (1999). Interpretive Criteria of Grated Exercise Test Results (Draft). *Chinese Journal of Cardiovascular Rehabilitation Medicine*.

[3] Liu JS, Yang JX, Zhang D (1994). Reference scheme of Chinese program for AMI cardiac rehabilitation. *Chinese Journal of Cardiovascular Rehabilitation Medicine*.

[4] Liu JS, Dai RZ, Cheng YZ, Guo L (2006). Chinese rehabilitation program after percutaneous coronary intervention (Trial version). *Chinese Journal of Cardiovascular Rehabilitation Medicine*.

[5] Hu DY (2015). Enduring twists and turns, the first glimpse of dawn. *Chinese Journal of Cardiovascular Medicine*.

[6] Chinese Society of Cardiology, Chinese Association of Rehabilitation Medicine Cardiovascular Disease Professional Committee, Gerontological Society of China Cardio-cerebrovascular Disease Professional Committee (2013). Expert consensus on coronary heart disease rehabilitation and secondary prevention in China. *Chinese Journal of Cardiology*.

[7] Chinese Association of Rehabilitation Medicine Cardiovascular Disease Professional Committee, Gerontological Society of China Cardio-cerebrovascular Disease Professional Committee (2014). Expert consensus on psychological prescriptions for patients with cardiovascular diseases in cardiovascular departments. *Chinese Journal of Cardiology*.

[8] Chinese Association of Rehabilitation Medicine Cardiovascular Disease Professional Committee, Clinical Nutrition Sub-committee of Chinese Nutrition Society, Chronic Disease Prevention and Control Branch of Chinese Preventive Medicine Association (2014). Expert consensus on nutritional prescriptions for cardiovascular diseases. *Chinese Journal of Internal Medicine*.

[9] Chinese Society of Cardiology Preventive Medicine Group, Chinese Association of Rehabilitation Medicine Cardiovascular Disease Professional Committee (2015). Expert consensus on exercise therapy for patients with coronary heart disease in China. *Chinese Journal of Cardiology*.

[10] Chinese Medical Doctor Association, Cardiovascular Medicine Physicians Branch, Prevention and Rehabilitation Professional Committee (2016). Expert Consensus on Exercise Rehabilitation After Percutaneous Coronary Intervention. *Chinese Journal of Interventional Cardiology*.

[11] Chinese Association of Rehabilitation Medicine, Heart Rehabilitation Professional Committee. (2016). Expert Consensus on Prescription Management of Stable Coronary Heart Disease Rehabilitation Drugs. *Chinese Journal of Cardiology*.

[12] Chinese Association of Rehabilitation Medicine Cardiovascular Disease Professional Committee. (2015). *Guidelines for Cardiac Rehabilitation and Secondary Prevention in China.*.

[13] Chinese Association of Rehabilitation Medicine Cardiovascular Disease Professional Committee. (2018). * Guidelines for Cardiac Rehabilitation and Secondary Prevention in China (2018 edition).*.

[14] National Center for Cardiovascular Diseases in China. (2016). * Expert Consensus on Integrated Chinese and Western Medicine Phase I Cardiac Rehabilitation.*.

[15] Clinical Pathways for Cardiovascular Disease Prevention and Rehabilitation. (2017). * Expert Consensus on Integrated Chinese and Western Medicine Phase I Cardiac Rehabilitation.*.

[16] Ding RJ, Hu DY (2018). China’s uneven development in cardiac rehabilitation services, with tremendous potential for future development of cardiac rehabilitation. http://acc2018.icirculation.com/newsview-32349-310-0.html.

[17] Chen Q, Li WH, Xie HZ, Chen H, Lu D (2021). A survey of the first stage Cardiac Rehabilitation in 76 tertiary hospitals in China. *Chinese Nursing Management*.

[18] Chinese Association of Rehabilitation Medicine, Cardiovascular Disease Prevention and Rehabilitation Professional Committee, China Association of Gerontology and Geriatrics, Cardiovascular Disease Professional Committee (2021). China expert consensus on center guided home-based cardiac rehabilitation. *Chinese Journal of Internal Medicine*.

[19] Zhang Z, Pack Q, Squires RW, Lopez-Jimenez F, Yu L, Thomas RJ (2016). Availability and characteristics of cardiac rehabilitation programmes in China. *Heart Asia*.

[20] Ding RJ, Lei S (2021). History, Current Situation and Countermeasure of Cardiac Rehabilitation in China. *Practical Journal of Cardiac Cerebral Pneumal and Vascular Diseases*.

[21] Gao W, Xu SL (2017). Historical review and academic development strategy of cardiac rehabilitation in China. *Zhonghua Xin Xue Guan Bing Za Zhi*.

[22] Guo Y, Ledesma RA, Peng R, Liu Q, Xu D (2017). The Beneficial Effects of Cardiac Rehabilitation on the Function and Levels of Endothelial Progenitor Cells. *Heart, Lung & Circulation*.

[23] Kong MY, Lu X, Lin S, Zhao Y, Lin AC (2017). Effects of physiological ischemia training on rabbits’ vascular endothelium during atherosclerotic plaque formation process. *Chinese Journal of Rehabilitation Medicine*.

[24] Zhao Y, Li J, Lin A, Xiao M, Xiao B, Wan C (2011). Improving angiogenesis and muscle performance in the ischemic limb model by physiological ischemic training in rabbits. *American Journal of Physical Medicine & Rehabilitation*.

[25] Yuan S, Xiao D, Zhou W, Li YJ (2019). High-intensity interval training followed by moderate intensity aerobic continuous exercise model for chronic heart failure. *Chinese Journal of Rehabilitation*.

[26] Gao ZZ, Ji P, Xia YQ, Wang L (2015). Effects of different intensity aerobic exercise on cardiac function and exercise endurance in patients after percutaneous coronary intervention. *Chinese Journal of Rehabilitation Medicine*.

[27] Dong L, Zhang Y, Liu SX (2018). Cardiovascular reaction in response to high-intensity interval training in healthy young men. *Chinese Journal of Hypertension*.

[28] Li BW, Tian L, Feng LL, Pan S, Tian ZJ (2023). Protective Effects of Whole-Body Vibration Training and High-Intensity Intermittent Exercise-on Cardiac Function and Skeletal Muscle in Rats with Myocardial Infarction by Up-Regulating MGF/MEK/ERK. *China Sport Science and Technology*.

[29] Zhou RF, Wu Q, Wang BZ (2023). Comparative study on the improvement of myocardial mitochondrial dysfunction by high-intensity interval aerobic exercise and moderate-intensity continuous aerobic exercise in myocardial infarction rats. *Prevention and Treatment of Cardio-Cerebral-Vascular Disease*.

[30] Gao W (2017). Clinical Study on Yang Xin Huo Xue Needling Method plus Western Medication for Chronic Heart Failure Due to Coronary Heart Disease. *Shanghai Journal of Acupuncture and Moxibustion*.

[31] Wang L, Li L (2019). Observation on Nursing Effect of Acupoint Application combined with Cardiac Rehabilitation in Treatment of Heart Failure. *Journal of Practical Traditional Chinese Internal Medicine*.

[32] Chen Q, Sun XL, Wan J, Hu TY (2023). The effect of early intervention based on the breath regulating of health Qigong Liuzijion on the cardiopulmonary function of patients with chronic heart failure. *Chinese Clinical Nursing*.

[33] Li SW, Yu ML, Gao X, Xu H, Chen KJ (2021). Effect of Sitting Baduanjin on Stage I Cardio-pulmonary Function of Patients after Coronary Artery Bypass Grafting. *Chinese Journal of Integrative Medicine on Cardio-Cerebrovascular Disease*.

[34] Wang JM, Liang C, Wang B, Pan DD, He ZQ, Li N (2018). Influence of “Sitting Baduanjin” on cardiac rehabilitation in patients with acute myocardial infarction after interventional treatment. *Chinese Journal of Integrative Medicine on Cardio-Cerebrovascular Disease*.

[35] Yu ML, Jiang H, Li B, Li B, Chai LL, Shen H (2020). Application of Tai Ji Quan Exercise in Heart Rehabilitation for Elderly Patients with Heart Failure after Myocardial Infarction. *Chinese Journal of Rehabilitation Theory and Practice*.

[36] Zheng JQ (2004). Observation on the rehabilitation effect of Tai Chi Quan on elderly coronary heart disease patients. *Chinese Journal of Rehabilitation Theory & Practice*.

[37] Liu XY, Huang JL, Li RM, Yang S (2022). Cardiac rehabilitation of patients with chronic heart failure based on Wuqinxi intervention. *Clinical Journal of Chinese Medicine*.

[38] Liu L (2020). Research on the application effect of Wuqinxi in cardiac rehabilitation of elderly patients. *Electronic Journal of Clinical Medical Literature*.

[39] Ke YW (2021). Clinical efficacy observation of Yijinjing in patients with chronic heart failure [master’s thesis]. *Fujian University of Traditional Chinese Medicine*.

[40] Su JJ, Wong AKC, Zhang LP, Bayuo J, Lin RS, Abu-Odah H (2023). Technology-assisted cardiac rehabilitation for coronary heart disease patients with central obesity: a randomized controlled trial. *European Journal of Physical and Rehabilitation Medicine*.

[41] Zheng TT, Li P, Lei J (2020). Feasibility of Wearable Motion Monitoring Bracelet in 6-minute Walking Test for Patients with Heart Disease. *China Continuing Medical Education*.

[42] Shi PH, Cao DH, Song AZ, Ye JB (2021). Influence of continuous follow-up using exercise wristbands on hope and self-efficacy in patients with myocardial infarction. *International Journal of Nursing*.

[43] Soroff HS, Hui J, Giron F (1986). Current status of external counterpulsation. *Critical Care Clinics*.

[44] Montalescot G, Sechtem U, Achenbach S, Andreotti F, Arden C, Task Force Members (2013). 2013 ESC guidelines on the management of stable coronary artery disease: the Task Force on the management of stable coronary artery disease of the European Society of Cardiology. *European Heart Journal*.

[45] International External Counterpulsation Society, Chinese Association of Rehabilitation Medicine Cardiovascular Disease Professional Committee, Gerontological Society of China Cardio-cerebrovascular Disease Professional Committee (2014). Cardiovascular disease rehabilitation prescription - Enhanced external counterpulsation application international expert consensus. *Chinese Journal of Internal Medicine*.

[46] Xiao YL, Li XG, Liu P, Li L, Zhang GQ (2016). Curative effect of external counterpulsation in treatment of coronary heart disease. *Chinese Journal of Evidence-Based Cardiovascular Medicine*.

[47] Sun G, Wu YH, Zhang XF, Tan WF, Zhang GX (2018). Short-term effect and safety of enhanced external counterpulsation on coronary flow after percutaneous coronary intervention in patients with unstable angina pectoris. *South China Journal of Cardiovascular Diseases*.

[48] Yang W, He Y, Gan L, Zhang F, Hua B, Yang P (2018). Cardiac shock wave therapy promotes arteriogenesis of coronary micrangium, and ILK is involved in the biomechanical effects by proteomic analysis. *Scientific Reports*.

[49] Yu W, Shen T, Liu B, Wang S, Li J, Dai D (2014). Cardiac shock wave therapy attenuates H9c2 myoblast apoptosis by activating the AKT signal pathway. *Cellular Physiology and Biochemistry: International Journal of Experimental Cellular Physiology, Biochemistry, and Pharmacology*.

[50] Liu B, Zhang Y, Jia N, Lan M, Du L, Zhao D (2018). Study of the Safety of Extracorporeal Cardiac Shock Wave Therapy: Observation of the Ultrastructures in Myocardial Cells by Transmission Electron Microscopy. *Journal of Cardiovascular Pharmacology and Therapeutics*.

[51] Liu WJ, Shen JY, Zhu MY, Zhang Y, Fan XH, Xu YW (2017). Efficacy and safety of extracorporeal cardiac shock wave therapy for intractable angina. *Shanghai Medical Journal*.

[52] LI Q, Yang J (2023). Research and Hot Topics of International Cardiac Rehabilitation: A Bibliometric Analysis. *Chinese Journal of Rehabilitation Medicine*.

[53] Qiu XF, Qiu C, Wang YL, Gao M (2018). Reliability and validity of Chinese version of Cardiac Rehabilitation Barriers Scale. *Academic Journal of Chinese PLA Medical School*.

[54] Liu X, Fowokan A, Grace SL, Ding B, Meng S, Chen X (2021). Translation, Cross-Cultural Adaptation, and Psychometric Validation of the Chinese/Mandarin Cardiac Rehabilitation Barriers Scale (CRBS-C/M). *Rehabilitation Research and Practice*.

[55] Xiao J, Huang LZ, Li LZ (2018). Development of a Chinese Version of Information Needs in Cardiac Rehabilitation Scale and Test of Its Reliability and Validity. *Chinese General Practice*.

[56] Ma C, Yang Q, Huang S (2019). Translation and Psychometric Evaluation of the Chinese Version of the Information Needs in Cardiac Rehabilitation Tool. *Journal of Cardiopulmonary Rehabilitation and Prevention*.

[57] Wang JH, Zhang ZX, Yang QF, Mei YX, Wang P (2019). Translation and reliability and validity of the Chinese version of the Cardiac Rehabilitation Inventory. *Chinese Nursing Journals Publishing House*.

[58] Zhang MN, Wang CM (2012). Study on testing of cardiac diet self-efficacy scale in the aged crpwd. *Chinese Nursing Research*.

[59] Chair SY, Wong KB, Tang JYM, Wang Q, Cheng HY (2015). Social support as a predictor of diet and exercise self-efficacy in patients with coronary artery disease. *Contemporary Nurse*.

[60] Sun YX, Zhao CY, Zhu Y, Yang ZY, Tang BX, Chen J (2021). The Chinesization and reliability and validity test of the Cardiac Exercise Self-Efficacy Instrument. *Chinese Journal of Modern Nursing*.

[61] Jiang W, Chen XK, Ding RJ, Fan ZQ, Hu DY (2022). Advantages of Chinese traditional exercise in heart rehabilitation. *Chinese Journal of General Practitioners*.

